# One Surgery, Two Solutions: A Systematic Review of Combined Autologous Breast Reconstruction and Lymphatic Surgery

**DOI:** 10.3390/curroncol33060338

**Published:** 2026-06-06

**Authors:** Ion Lingenheil, Lisa Radacher, Hans-Günther Machens, Michael Mayr-Riedler, Katrin Seidenstücker, Niclas Peter Broer, Lisanne Grünherz

**Affiliations:** 1 Department of Plastic, Reconstructive, Aesthetic, Hand- and Burn Surgery, Munich Clinic Bogenhausen, 81925 Munich, Germanylisa.radacher@gmx.at (L.R.);; 2 Department of Plastic and Hand Surgery, Klinikum Rechts der Isar, Technical University Munich, 81675 Munich, Germany; 3 Department for Plastic and Reconstructive Surgery, Sana Kliniken Düsseldorf, 40625 Düsseldorf, Germany; katrin.seidenstuecker@sana.de; 4 School of Medicine and Health, Technical University Munich, 81675 Munich, Germany

**Keywords:** autologous breast reconstruction, lymphatic reconstruction, vascularized lymph node transfer, lymphovenous anastomosis

## Abstract

Simultaneous autologous breast reconstruction (ABR) and lymphatic surgery has emerged as a strategy to address breast cancer-related lymphedema (BCRL) while restoring breast contour within a single operative procedure. In light of the diversity of surgical strategies, we aimed to evaluate the current literature on combined ABR and lymphatic surgery, with particular focus on surgical techniques, clinical outcomes, complications, and patient-reported satisfaction. A systematic review was conducted according to PRISMA guidelines and registered in PROSPERO (CRD420251135446). Medline, Embase, CENTRAL, Web of Science, and PubMed were searched through 9 January 2026 Studies reporting outcomes of simultaneous ABR and lymphatic reconstruction were included. Data on surgical techniques, complications, changes in limb volume and cellulitis incidence, and patient-reported outcomes were extracted. Twenty-seven studies including 499 patients (mean follow-up 23 months) were analyzed. The most common approach was a chimeric deep inferior epigastric perforator (DIEP) flap with inguinal lymph nodes (459 patients), followed by ABR with a separate vascularized lymph node transfer and ABR with lymphovenous anastomosis. Most studies reported postoperative reductions in limb volume and cellulitis, with cellulitis reduction rates up to 100%. Patient-reported outcomes (LYMQOL, ULL-27, LYMPH-Q) showed improved quality of life. Complication rates were low, including 4% seroma and 1% flap loss after chimeric DIEP. Simultaneous ABR and lymphatic reconstruction is feasible and associated with improved clinical and patient-reported outcomes. However, heterogeneity limits comparison between the different surgical techniques, and prospective studies with standardized outcomes measurements are needed.

## Simple Summary

This systematic review looked at a surgical approach that combines breast reconstruction using a patient’s own tissue with procedures to improve lymph fluid drainage after breast cancer treatment. It included 27 studies, with 499 patients followed for about two years. The most common method used tissue from the lower abdomen along with lymph nodes to rebuild the breast and help reduce swelling in the arm. Overall, patients showed reduced arm swelling, fewer infections and improved quality of life. Complications were uncommon, with small fluid collections occurring in a few cases and very rare loss of the reconstructed tissue. In general, combining these procedures appears to be a safe and effective option, although more consistent and long-term research is still needed.

## 1. Introduction

Autologous breast reconstruction (ABR) and lymphatic surgeries, such as lymphovenous anastomosis (LVA) and vascularized lymph node transfer (VLNT), have emerged as effective procedures to address both the physical and psychological sequelae of breast cancer treatment. In recent years, there has been growing interest in combining ABR with lymphatic surgery in a single operative session, aiming to restore breast contour while simultaneously treating or preventing breast cancer-related lymphedema (BCRL) [1,2]. Despite an increasing number of reports describing this combined approach, the comparative effectiveness, safety, and long-term outcomes of the different surgical techniques and combinations have not been systematically synthesized.

Over the past few decades, significant advances have been made in the oncological management of breast cancer. Improved screening methods and more effective systemic therapies have led to a steady decline in the need for mastectomy and axillary lymph node dissection. In parallel, an increasing proportion of patients can be treated with breast-conserving surgery and sentinel lymph node biopsy alone [3,4]. Moreover, novel surgical techniques have been developed to mitigate the risk of arm lymphedema, even in patients undergoing axillary lymph node dissection. One such approach is the Lymphatic Microsurgical Preventive Healing Approach (LYMPHA), in which one to four LVAs are performed at the time of axillary lymph node removal. While several studies have reported that patients undergoing LYMPHA did not develop arm lymphedema [5,6,7], the overall benefit of this approach remains questionable. Notably, a prospective case–control study demonstrated no significant difference in lymphedema development after axillary dissection with or without concomitant lymphatic reconstruction [8].

Despite these advances, the incidence of upper extremity lymphedema following axillary lymph node dissection combined with radiotherapy remains as high as 30% and is well known to be associated with a substantial impairment in arm function [9,10]. Both the loss of the breast following mastectomy and the development of arm lymphedema result in profound physical and psychological distress [11,12,13]. In this context, combined ABR and lymphatic reconstruction represents a promising surgical strategy. This systematic review therefore aims to evaluate the current literature on combined ABR and lymphatic surgery, with particular attention to surgical techniques, clinical outcomes, complications, and patient-reported satisfaction.

## 2. Materials and Methods

We performed a systematic review in accordance with the Preferred Reporting Items for Systematic Reviews and Meta-Analysis (PRISMA) guidelines. A review protocol was designed and registered on PROSPERO, the international prospective register of systematic reviews (Registration number: CRD420251135446). A comprehensive literature search was conducted on 9 January 2026, across multiple databases, including Medline, Embase, Central Cochrane, Web of science and PubMed. Language was restricted to English and German. There was no time restriction. The search strategy employed a combination of controlled vocabulary terms and free-text terms, which were adapted for each database. Details of the individual search equations for all databases are provided as Supplementary Table S1.

The resulting records were imported into Rayyan, where duplicates were removed. Two authors (IL, LG) independently screened the titles and abstracts of the retrieved studies. Discrepancies were resolved by a third reviewer (LR). Full-text articles of potentially relevant studies were then reviewed for eligibility, with all disagreements ultimately resolved by consensus among the three reviewers.

Inclusion criteria were limited to randomized controlled trials, prospective cohort studies, retrospective cohort studies, cross-sectional studies, case-reports and case series. Patients were required to have undergone simultaneous microvascular breast and lymphatic reconstruction. Systematic reviews, meta-analyses, and publications not written in English were excluded, as well as cadaveric studies and papers introducing novel techniques without defined outcome measurements, e.g., volume measurements.

For quantitative analysis the following parameters were extracted: number of patients, age, lymphedema stage, circumference/volume measurements, complications and patient-reported outcome measurements. When studies compared simultaneous breast and lymph reconstruction with other techniques, e.g., lymphatic reconstruction alone, data for the qualifying groups was extracted separately and included in the analysis.

The methodological quality of the included studies was evaluated using the Methodological Index for Non-Randomized Studies (MINORS) instrument. Additionally, the completed PRISMA checklist is provided as Supplementary Material File S1 [14]. 

## 3. Results

**Figure 1:**
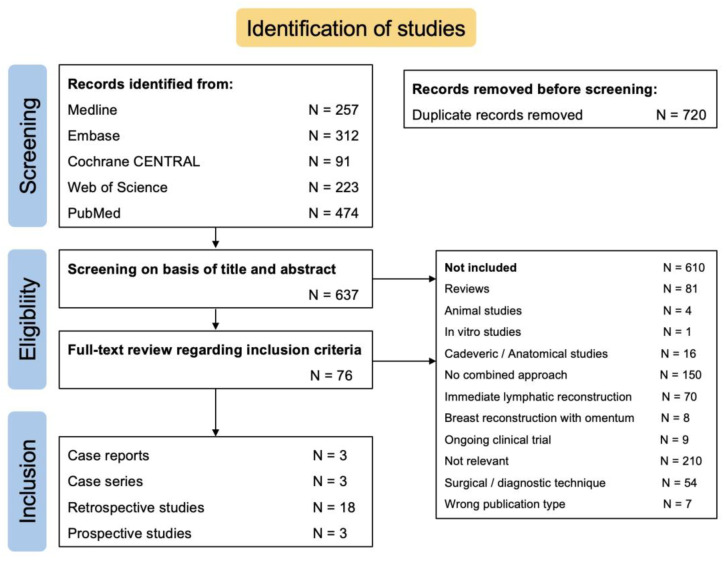
PRISMA Flowchart demonstrating the selection process of studies.

The systematic search yielded 637 articles after removal of duplicates. Following review of title and abstract, 76 articles were included in full-text review. Based on the inclusion criteria, 27 articles were finally selected. The detailed selection process is shown in Figure 1.

Of the 27 included studies, 23 could be evaluated using the MINORS instrument. The remaining studies were excluded from the quality assessment because they were case reports or case series with insufficient methodological information. As most studies did not include a comparative cohort, the 8-item version of the MINORS scale was used. The median score was 6, indicating overall limited methodological quality of the available evidence. (Supplementary Table S2).

Across all studies, 499 patients undergoing simultaneous microvascular breast and lymphatic reconstruction with a mean follow-up of 23 months were analyzed. The mean age at the time of surgery was 51 years. Most patients were diagnosed with lymphedema stage 2 according to the staging of the International Society of Lymphology (ISL) while only a very small proportion of patients received prophylactic lymphatic reconstruction after axillary lymph node dissection. We identified the following combinations of surgical therapy: Chimeric deep inferior epigastric perforator (DIEP) with inguinal lymph nodes, ABR and VLNT as separate flaps and ABR combined with LVAs.

### 3.1. Chimeric Deep Inferior Epigastric Perforator Flap with Inguinal Lymph Nodes

**Figure 2:**
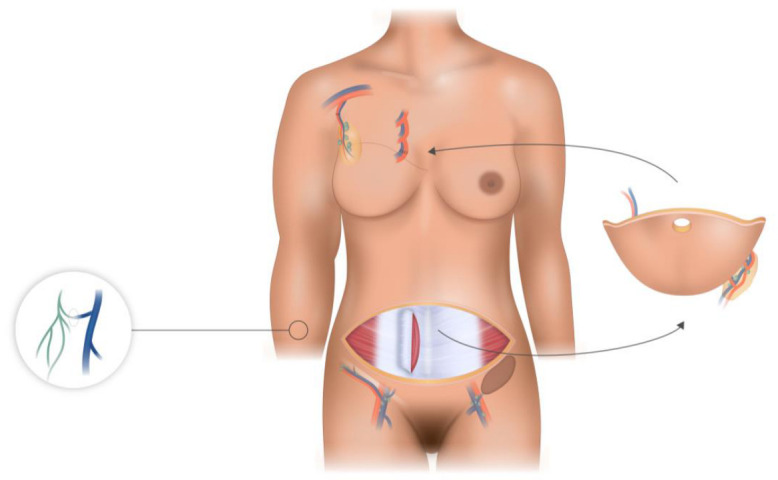
A chimeric DIEP flap is harvested together with inguinal lymph nodes based on the superficial inferior epigastric or superficial circumflex iliac vessels. The inferior epigastric artery and vein are then anastomosed to the internal mammary vessels. An additional arterial and venous anastomosis may be performed between the pedicle supplying the lymph nodes and the thoracodorsal vessels. The affected arm may be further treated with lymphovenous anastomosis.

In more than half of the relevant studies a chimeric DIEP flap with incorporation of inguinal lymph nodes was harvested (Figure 2). We identified 21 studies, comprising a total of 459 patients with a mean follow-up of 25 months (Table 1). The majority of women were diagnosed with stage II arm lymphedema. In all studies the lymph nodes were harvested along the superficial epigastric inferior or superficial circumflex iliac vessels. Only three studies reported the number of lymph nodes, with a range of 3 to 5 nodes [15,16,17]. In four studies surgeons performed additional LVAs [2,16,18,19].

Most authors base their flap design on preoperative computed tomography angiography. In addition, in the majority of studies preoperative lymphoscintigraphy and intraoperative reverse lymph node mapping was performed to identify and spare sentinel lymph nodes in the groin. While the DIEP flap was anastomosed to the internal mammary vessels in most of the studies with a few exceptions with anatomosis to the thoracodorsal vessles, there was considerable heterogenity with regard to the revascularization of the lymph node flap at the axilla. In six studies, both the arterial and venous pedicles were anastomosed to the thoracodorsal vessels or to branches of the serratus or lateral thoracic wall. The remaining authors performed either venous supercharging alone or no additional arterial or venous anastomoses when the lymph node flap demonstrated adequate perfusion [31].

Based on seven studies the mean postoperative reduction in cellulitis episodes is 84% (range: 50–100%). While 14 out of 21 studies reported postoperative improvements in arm volume, there was considerable heterogeneity in the methods used to assess volume and circumference, limiting the comparability of results across studies.

### 3.2. Autologous Breast Reconstruction and Separate Vascularized Lymph Node Transfer

**Figure 3:**
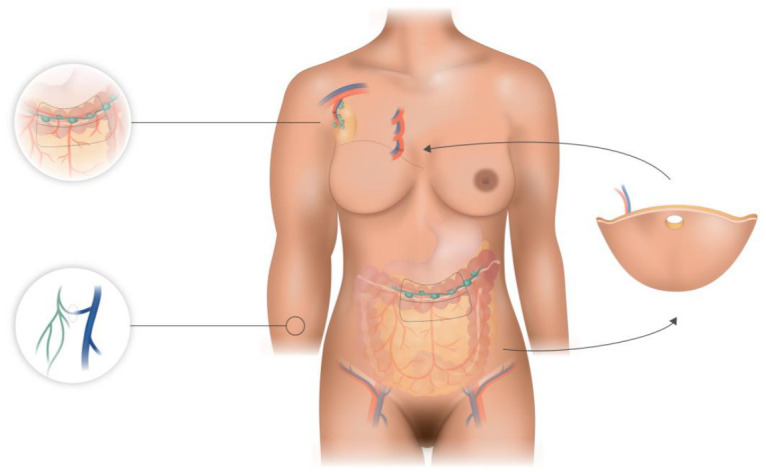
A DIEP flap is harvested and anastomosed to the internal mammary vessels. During the same procedure, a gastroepiploic lymph node flap is harvested laparoscopically and anastomosed to the thoracodorsal vessels at the axilla. The affected arm may additionally undergo lymphovenous anastomosis to further enhance lymphatic drainage.

Eight studies on simultaneous microvascular ABR based on a DIEP flap and a separate VLNT were identified, comprising a total of 52 patients with a mean follow-up of 22 months and the majority presenting with stage II arm lymphedema (Table 2). In five of these studies, lymph nodes were harvested from the omentum as gastroepiploic vascularized lymph node flap (Figure 3) [34,35,36,37], while the remaining study utilized submental or inguinal VLNT [38]. In all studies, the DIEP flap was anastomosed to the internal mammary vessels. Depending on the VLNT recipient site, microvascular anastomosis was performed at the axilla, proximal forearm, or wrist. None of the studies performed simultaneous LVA.

Based on two retrospective studies, Dionyssiou described a pedicled, fat-augmented latissimus dorsi flap combined with simultaneous VLNT as an alternative for thin, nulliparous patients. In this technique, lymph nodes were harvested either from the ipsilateral lateral thoracic wall or from the groin and revascularized to the lateral thoracic or serratus anterior vessels [15,17].

Based on three studies, the postoperative cellulitis reduction rate ranged from 69% to 83% [15,36,38]. Similarly, analysis of four studies showed a postoperative circumference reduction rate ranging between 25% and 56% [34,36,37,38].

### 3.3. Autologous Breast Reconstruction and Lymphovenous Anastomosis

**Figure 4:**
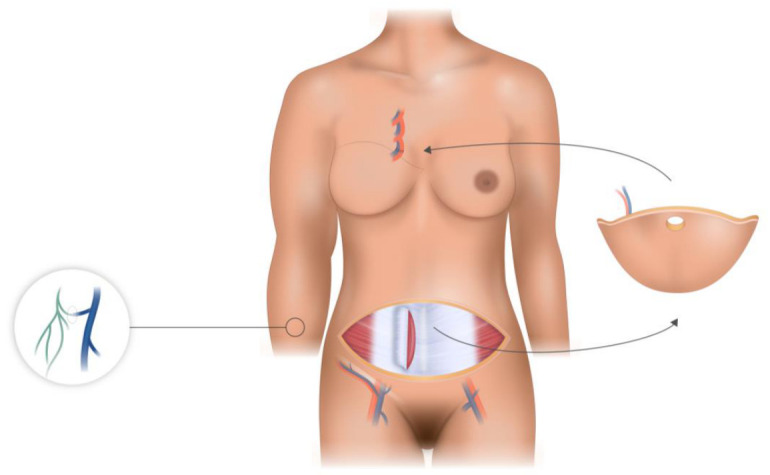
A DIEP flap is harvested and microsurgically anastomosed to the internal mammary vessels. Concurrently, lymphovenous anastomoses are performed in the affected arm to enhance lymphatic drainage.

We identified only one study [38] that performed ABR based on DIEP or PAP flap with simulatenous LVAs of the affected arm (Figure 4). Analogous to the publications mentioned above, LVAs were created following prior intraoperative lymphography in four patients with lymphedema grade I and II according to the Cheng classification. Based on the comparison of the postoperative circumference reduction rate, the authors demonstrate that ABR and LVAs resulted in less volume reduction than ABR combined with VLNT.

### 3.4. Complications

**Figure 5:**
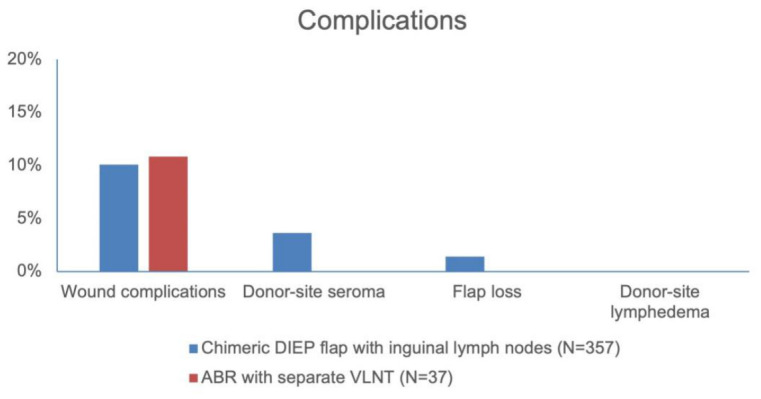
Bar chart showing the distribution of complications after chimeric DIEP flap with inguinal lymph nodes compared with autologous breast reconstruction (ABR) with a separate vascularized lymph node transfer (VLNT).

For the quantitative analysis of complications, eleven studies on chimeric DIEP flaps with inguinal lymph nodes and six studies on ABR with separate VLNT were included. Wound complications were the most common adverse events in both groups (Figure 5). Seroma at the lymph node harvest site occurred in 4% of chimeric DIEP cases, and flap loss in 1%. No donor-site lymphedema was reported in either group.

### 3.5. Patient-Reported Outcome Measurements

Overall, five studies included one of the following Patient-reported outcome measurements (PROMS) that have been well validated for arm lymphedema: ULL-27, LYMPH-Q and LYMQOL. All of these studies evaluated patients who underwent a chimeric DIEP flap combined with inguinal lymph nodes and could demonstrate significant improvements in different domains of quality of life. Briefly, the ULL-27 was used in two studies comprising a total of 70 patients, in which significant improvements were observed across the physical, psychological, and social domains. The LYMQOL was assessed in three studies, comprising a total of 66 patients, demonstrating significant improvements across all domains, including appearance, function, symptoms, mood, and overall score.

Myung et al. evaluated the LYMPH-Q in a cohort of 23 patients who underwent a muscle-sparing TRAM flap with simultaneous VLNT. The LYMPH-Q comprises six distinct domains; however, instead of reporting each domain separately, the authors calculated a composite mean score across all domains. This averaged score showed significant improvements at 12 months postoperatively, indicating an overall enhancement in quality of life.

## 4. Discussion

This systematic review demonstrates that simultaneous microvascular breast and lymphatic reconstruction is a feasible and increasingly utilized approach for the management of breast cancer-related lymphedema, with a growing body of evidence supporting its effectiveness. Across the included studies, most patients underwent chimeric DIEP flap reconstruction incorporating inguinal lymph nodes, whereas alternative strategies, including microvascular ABR with simultaneous VLNT or combined with LVAs, were less frequently reported.

The available data confirm postoperative improvements in arm volume, reductions in cellulitis rates and patient-reported quality of life. With regard to volume and circumference measurements all studies reported on postoperative improvements. Due to a high heterogeneity across studies a more detailed quantitative analysis regarding extremity volume in relation to different surgical techniques, was not feasible. Therefore, conclusions can only be drawn based on the frequency of reported approaches in the literature. Most authors reported the use of a chimeric DIEP flap combined with inguinal lymph nodes, which offers the advantage of a single donor site and a single microsurgical flap harvest. This approach may reduce operative complexity and shorten surgical time, particularly when compared with techniques requiring the harvest of a second flap for lymphatic reconstruction.

Interestingly, we observed considerable heterogeneity in the revascularization strategies of the lymph node component. While the DIEP flap itself was consistently anastomosed to the internal mammary vessels, management of the lymph node flap varied substantially, ranging from complete arterial and venous revascularization to venous supercharging alone, or even omission of additional microvascular anastomoses when flap perfusion appeared clinically adequate. Although this aspect has not yet been systematically analyzed, such variability may have important implications for outcomes following lymphatic reconstruction.

Complete arterial and venous revascularization of the lymph node flap may theoretically optimize tissue perfusion, lymphangiogenesis, and the long-term viability of transferred lymphatic tissue. Adequate arterial inflow may be particularly important for maintaining the metabolic activity of transferred lymph nodes and supporting the release of lymphangiogenic growth factors, such as vascular endothelial growth factor-C, which are believed to promote regeneration of lymphatic channels [40]. Similarly, the importance of sufficient venous drainage in VLNT has increasingly been emphasized in the literature. Ciudad et al. described venous hypertension as a potential limitation of vascularized lymph node flaps and proposed venous supercharging to optimize flap hemodynamics and reduce inflow–outflow mismatch [41]. Furthermore, given that lymphatic fluid is ultimately transferred into the venous circulation through intrinsic lymphovenous communications, optimization of venous outflow may not only improve flap perfusion but also directly enhance the functional efficacy of lymphatic reconstruction [42]. These considerations may explain why some authors attribute increased emphasis to an additional venous anastomosis of the included lymph node flap.

The underlying lymphatic mechanisms and the functional integration of transferred lymph nodes remain incompletely understood. In particular, it remains unclear to what extent transferred lymph nodes establish effective drainage toward the recipient basin or the arm. Five studies performed lymphoscintigraphy at the 12-month follow-up, confirming radiotracer uptake in the axilla and improved clearance through the lymphatic channels [15,17,27,29,31]. Similar findings were observed on postoperative magnetic resonance lymphangiography, providing further evidence of reconnection between afferent lymphatic channels and the recipient lymphatic system [43]. However, some authors observed improvements in edema limited to the breast and the lateral thoracic wall, without a corresponding reduction in arm circumference. As a result, they modified their surgical approach to a staged strategy, separating autologous breast reconstruction using a DIEP flap from lymphatic reconstruction [44].

The rate of seroma formation following chimeric DIEP with inguinal lymph nodes was notably low, particularly given that groin lymph node harvest is associated with a seroma risk of up to 60% [45,46], while DIEP flap harvest itself carries a reported incidence of up to 48% [47]. This finding is likely attributable to the adoption of refined surgical techniques aimed at minimizing seroma formation, including progressive tension sutures, meticulous ligation of lymphatic vessels, and reduction in dead space in the groin [46]. Additionally, some surgeons preserve extra subcutaneous tissue on the superior skin flap at the level of lymph node harvest site [25,28]. During abdominal closure, this tissue is transposed to fill the resulting defect in the groin, thereby reducing dead space and potentially lowering the risk of postoperative fluid accumulation.

In six studies, health-related QOL was assessed using validated PROMs. Notably, all patients in these studies underwent chimeric DIEP flap reconstruction with inguinal lymph nodes. Consistent with a growing body of evidence demonstrating improved QOL following lymphatic reconstruction [45,48,49], significant improvements across the various domains of the LYMPH-Q, ULL-27, and LYMQOL indicate postoperative enhancements in physical functioning, psychological well-being, social participation, and symptom burden. Interestingly, Winters et al. performed an additional correlation analysis and found a significant moderate correlation between improvements in QOL, as measured by the total score of the ULL-27, and preoperative physiotherapy, as well as a postoperative reduction in physiotherapy and compression garment use [1]. Furthermore, the study by Myung et al. suggests greater improvements in QOL following ABR combined with VLNT compared with VLNT alone [2]. However, these findings should be interpreted with caution, as ABR itself has a substantial impact on body image and appearance, which may have independently influenced the results of the different QOL scales.

This systematic review is limited by the substantial heterogeneity among the included studies, which restricts direct comparability and precludes further quantitative analysis. Differences in surgical techniques, adjunctive procedures, outcome measurements, and follow-up protocols contributed to considerable variability across studies. In addition, most studies were retrospective in design, of low methodological quality, and involved relatively small patient cohorts, increasing the risk of selection and reporting bias. Several studies also originated from the same author groups, raising the possibility of partially or completely overlapping patient populations. The results of the quality assessment have now been added to the manuscript and are presented in Supplementary Table S2. We also acknowledge in the discussion that the predominance of retrospective and non-comparative studies limits the strength of the conclusions that can be drawn.

## 5. Conclusions

In conclusion, simultaneous ABR with lymphatic reconstruction represents a feasible and increasingly applied surgical strategy for BCRL, with consistent improvements in limb volume, cellulitis incidence, and patient-reported outcomes. However, substantial heterogeneity in study design, outcome reporting, and surgical techniques limits direct comparability and precludes further quantitative synthesis. While most surgeons support the use of chimeric DIEP flaps incorporating inguinal lymph nodes, the optimal surgical strategy and the underlying mechanisms governing lymphatic function and arm drainage after transfer remain incompletely understood. Further prospective studies with objective lymphatic imaging and long-term follow-up are required to refine surgical techniques and clarify the mechanism of lymphatic integration.

## Figures and Tables

**Table 1:** Studies on chimeric DIEP flap with inguinal lymph nodes.

Author (Year)	Study Design	N	ISL Stage (N)	Recipient Vessels VLNT	Additional LVA Mean (N)	Outcome Lymphedema (Volume/Circumference)	Cellulitis Reduction %	QOL Instrument
Szymanski (2024) [20]	Case report	1	Stage 1, Stage 2	Retrograde IMV		NA	NA	NA
Aktia (2016) [21]	Case Report	1	NA	LTA + LTV or SBA + SBV		NA	NA	NA
Akita (2017) [22]	Retrospective	13 (27) #	NA	NA	NA	NA	NA	NA
Parikh (2021) [23]	Case series	5	NA	IM (additional intraflap anastomosis)		in 80% of patients LE completely resolved, 20% improved	NA	NA
Demiri (2024) [24]	Retrospective	34	Stage 1 (10); Stage 2 (21); Stage 3 (3)	TDA + TDV		17.5% mean volume differential ratio	NA	self-evaluation questionnaires
Myung (2023) [2]	Retrospective	23 (87) #	Stage 2b (11); Stage 3 (76)	NA	1.3	decreased from 1.3 to 1.2 times larger than contralateral limb	NA	LYMPH-Q
Dionyssiou (2021) [17]	Retrospective	18 (64) #	Stage 1 (11); Stage 2 (34); Stage 3 (19)	TDA + TDV or LTA + LTV		63% mean volume differential reduction	NA	NA
Dionyssiou (2022) [15]	Retrospective	24 (69) #	Stage 1 (23); Stage 2 (36); Stage 3 (10)	TDA + TDV or LTA + LTV		54.8% mean volume reduction	83.3	VAS
Chang (2020) [16]	Retrospective	38	NA	TDA + TDV or LTA + LTV (if necessary)	1.53	81.6% had a reduction in volume	100	NA
Di Taranto (2023) [25]	Retrospective	32	Stage 2	LTV or SBV (no additional artery)		improved circumference reduction rate	100	LYMQOL + BREAST-Q
Winters (2022) [1]	Retrospective	64	Stage 1 (15); Stage 2 (22)	NA		no volume difference	85.7	ULL-27
De Brucker (2016) [26]	Retrospective	22	Stage 1, Stage 2	LTA + LTV or SBA + SBV		NA	50	ULL-27
Nguyen (2015) [27]	Retrospective	29	NA	TDA + TDV or LTA + LTV or SBA + SBV		10% improvement in volume differential	NA	NA
Dancey (2013) [28]	Retrospective	18	NA	no additional anastomosis		NA	NA	LYMQOL
Saaristo (2012) [29]	Retrospective	9 (78) #	NA	retrograde to TDA + TDV (if necessary)		77.7% had a reduction in volume	NA	NA
Wallis (2019) [30]	Retrospective	16	NA	NA		NA	NA	LYMQOL
Chen (2014) [31]	Retrospective	10	NA	no additional anastomosis		88.9% had a reduction in volume	NA	NA
Chu (2023) [19]	Retrospective	3	NA	NA	1.5	11.8% volumetric reduction (perometer)	100	NA
Chang (2018) [32]	Prospective	30 (57) #	NA	NA	2	76.4% volumetric reduction (perometer)	NA	NA
Chang (2020) [18]	Prospective	33 (54) #	NA	LTV or SBV (no additional artery)	1.5	60% volumetric reduction (perometer)	100	NA
Montag (2019) [33]	Prospective	15 (24) #	Stage 1 (3); Stage 2 (15); Stage 3 (6)	TDA + TDV or LTA + LTV		20.6% mean volume loss	70	NA

NA—not available; VLNT—vascularized lymph node transfer; LVA—lymphovenous anastomosis; TDA—thoracodorsal artery; TDV—thoracodorsal vein; IMV—internal mammary vein; LTA—lateral thoracic artery; LTV—lateral thoracic vein; URV—ulnar recurrent artery and vena commitantes; RA—radial artery and vena commitantes; SBA—arterial branch to serratus; SBV—venous branch to serratus. # Number in brackets refers to the total study population; only a subgroup received the combined approach analyzed.

**Table 2:** Studies on autologous breast reconstruction and separate vascularized lymph node transfer.

Author (Year)	Study Design	N	ISL Stage (N)	Surgical Technique	Recipient Vessels VLNT	Outcome	Cellulitis Reduction Rate
Ciudad (2022) [36]	Case report	1	prophylactic	DIEP + GE-VLNT	LTA + LTV	no increase in circumference	NA
Crowley (2024) [35]	Case series	7	Stage 1 (2); Stage 2 (4); Prophylactic (3)	DIEP /MSTRAM + GE-VLNT	URV/RV	NA	NA
Ciudad (2020) [37]	Case series	6	Stage 2b–3	DIEP + GE-VLNT	RA/CV	30% circumference reduction rate	NA
Ciudad (2023) [34]	Retrospective	10 (78) #	Stage 2 (40); Stage 3 (38)	DIEP + GE-VLNT	LTA + LTV	56% circumference reduction rate	80% (in ISL stage 2)
Engel (2018) [38]	Retrospective	11 (124) #	NA	DIEP + submental or inguinal VLNT	NA	24.9% circumference reduction rate	69%%
Deldar (2017) [39]	Retrospective	5	NA	DIEP + GE-VLNT	NA	66.34% volumetric reduction rate	NA
Dionyssiou (2021) [17]	Retrospective	4 (64) #	Stage 1 (11); Stage 2 (34); Stage 3(19)	fat augmented LT + lateral thoracic wall lymph nodes or groin VLNT	LTA + LTV or SBA + SBV	63% mean volume differential reduction	NA
Dionyssiou (2022) [15]	Retrospective	8 (69) #	Stage 1 (23); Stage 2 (36); Stage 3 (10)	fat augmented LT + lateral thoracic wall lymph nodes or groin VLNT	LTA + LTV or SBA + SBV	54.8% mean volume reduction	83%

NA—not available; DIEP—deep inferior epigastric perforator flap; GE-VLNT—gastroepiploic vascularized lymph node transfer; VLNT—vascularized lymph node transfer; LT—latissimus dorsi; LTA—lateral thoracic artery; LTV—lateral thoracic vein; URV—ulnar recurrent artery and vena commitantes; RA—radial artery and vena commitantes; SBA—arterial branch to serratus; SBV—venous branch to serratus. # Number in brackets refers to the total study population; only a subgroup received the combined approach analyzed.

## Data Availability

No new data were created or analyzed in this study. Data sharing is not applicable to this article.

## Supplementary Materials

The following supporting information can be downloaded at: https://www.mdpi.com/article/10.3390/curroncol33060338/s1, Supplementary Table S1: Search equations for all databases; Supplementary Table S2: MINORS score; File S1: PRISMA 2020 Checklist. Reference [14] has been cited in Supplementary Materials.

## Abbreviations

The following abbreviations are used in this manuscript:

PRISMA	Preferred Reporting Items for Systematic Reviews and Meta-Analysis
ABR	Autologous breast reconstruction
BCRL	Breast cancer related lymphedema
VLNT	vascularized lymph node transfer
GE-VLNT	gastroepiploic vascularized lymph node transfer
LVA	lymphovenous anastomosis
DIEP	deep inferior epigastric perforator
MSTRAM	muscle sparing transverse rectus abdominis muscle
ULL-27	Upper Limb Lymphedema 27
LYMQOL	Lymphedema Quality of Life Questionnaire
QOL	quality of life
PROMs	patient reported outcome measurements
ISL	International society of lymphology
TDA	thoracodorsal artery
TDV	thoracodorsal vein
IMV	internal mammary vein
LTA	lateral thoracic artery
LTV	lateral thoracic vein
URV	ulnar recurrent artery and vena commitantes
RA	radial artery and vena commitantes
SBA	arterial branch to serratus
SBV	venous branch to serratus
NA	not available
