# Supplementary material for: One Surgery, Two Solutions: A Systematic Review of Combined Autologous Breast Reconstruction and Lymphatic Surgery
Source: Curr Oncol. 2026 Jun 6;33(6):338. doi: 10.3390/curroncol33060338 (PMC13297874; doi:10.3390/curroncol33060338)
Supplement: Supplementary file 1 [file curroncol-33-00338-s001.zip › supplementary tables.pdf]

Table S1. Search equations for all databases

Concept	MeSH-Terms / Emtree-Terms	Key Words (Title/Abstract)
**Autologous Breast reconstruction**	"Mammaplasty"[MeSH] OR "Breast Reconstruction"[MeSH]	breast reconstruction OR autologous breast reconstruction<br>OR DIEP<br>OR DIEAP<br>OR SIEA<br>OR TRAM<br>OR SGAP<br>OR PAP<br>OR TMG<br>OR TUG<br>OR latissimus dorsi flap<br>OR free flap
**Reconstructive Lymphatic surgery**	"Lymphatic Vessels/surgery"[MeSH] OR "Lymph Node Transfer"[MeSH] OR "Lymphatic Anastomosis, Surgical"[MeSH]	lymphatic surgery OR<br>lymphatic reconstructive surgery OR lymphovenous anastomosis OR lymphovenous bypass OR LVA OR vascularized lymph node transfer OR VLNT OR lymph node flap OR lymphatic reconstruction<br>OR gastroepiploic lymph node transfer OR lymph node transplantation
**Breast cancer / mastectomy context**	"Breast Neoplasms"[MeSH]	breast cancer OR breast carcinoma OR mastectomy OR postmastectomy
**Breast-Cancer related Lymphedema**	"Lymphedema"[MeSH] OR "Lymphatic Diseases/surgery"[MeSH]	lymphedema OR lymphoedema OR breast cancer related lymphedema OR BCRL

Database	Download	Search string	Filter / Limits	Number of publications
**PubMed / MEDLINE**	09.01.2026	**Document 1:**<br>(("Mammaplasty"[MeSH Terms] OR "breast reconstruct*"[Title/Abstract] OR "autologous"[Title/Abstract] OR "microsurg*"[Title/Abstract] OR "microvascular"[Title/Abstract] OR "free flap"[Title/Abstract] OR "free tissue transfer"[Title/Abstract] OR "perforator flap*"[Title/Abstract] OR "DIEP"[Title/Abstract] OR "deep inferior epigastric perforator"[Title/Abstract] OR "TRAM"[Title/Abstract] OR "SIEA"[Title/Abstract] OR "SGAP"[Title/Abstract] OR "PAP"[Title/Abstract] OR "TMG"[Title/Abstract] OR "TUG"[Title/Abstract] OR "latissimus dorsi"[Title/Abstract] OR "LD flap"[Title/Abstract]) AND ("lymphatic vessels/surgery"[MeSH Terms] OR "lymphovenous"[Title/Abstract] OR "lymphaticovenous"[Title/Abstract] OR "lymphaticovenular"[Title/Abstract] OR "anastomos*"[Title/Abstract] OR "bypass"[Title/Abstract] OR "shunt"[Title/Abstract] OR "LVA"[Title/Abstract] OR "VLNT"[Title/Abstract] OR "vascularized lymph node"[Title/Abstract] OR "lymph node transfer"[Title/Abstract] OR "lymph node flap"[Title/Abstract] OR "immediate lymphatic reconstruction"[Title/Abstract] OR "ILR"[Title/Abstract] OR "LYMPHA"[Title/Abstract] OR "supermicrosurg*"[Title/Abstract]) AND ("Breast Neoplasms"[MeSH Terms] OR "breast cancer"[Title/Abstract] OR "breast carcinoma"[Title/Abstract] OR "mastectom*"[Title/Abstract] OR "postmastectom*"[Title/Abstract])) AND ((humans[Filter]) AND (english[Filter]) AND (2000:2026[pdat]))<br><br>**Document 2:**<br>("Breast Reconstruction"[MeSH] OR "Mammaplasty"[MeSH] OR breast reconstruct*[tiab] OR autologous[tiab] OR microsurg*[tiab] OR microvascular[tiab] OR "free flap"[tiab] OR "free tissue transfer"[tiab] OR perforator flap*[tiab] OR DIEP[tiab] OR "deep inferior epigastric perforator"[tiab] OR TRAM[tiab] OR SIEA[tiab] OR SGAP[tiab] OR PAP[tiab] OR TMG[tiab] OR TUG[tiab]	Humans, English, 2000–current	n = 328 (Dok1) and 146 (Dok2)

		OR "latissimus dorsi"[tiab] OR "LD flap"[tiab]) AND ("Lymphatic Vessels/surgery"[MeSH] OR "Lymph Node Transfer"[MeSH] OR "Lymphatic Anastomosis, Surgical"[MeSH] OR lymphovenous[tiab] OR lymphaticovenous[tiab] OR lymphaticovenular[tiab] OR anastomos*[tiab] OR bypass[tiab] OR shunt[tiab] OR LVA[tiab] OR VLNT[tiab] OR "vascularized lymph node"[tiab] OR "lymph node transfer"[tiab] OR "lymph node flap"[tiab] OR "immediate lymphatic reconstruction"[tiab] OR ILR[tiab] OR LYMPHA[tiab] OR supermicrosurg*[tiab]) AND ("Breast Neoplasms"[MeSH] OR breast cancer[tiab] OR breast carcinoma[tiab] OR mastectom*[tiab] OR postmastectom*[tiab]) AND ("Lymphedema"[MeSH] OR lymphedema[tiab] OR lymphoedema[tiab] OR BCRL[tiab] OR "breast cancer-related lymphedema"[tiab] OR secondary lymphedema[tiab])		
**Embase.com**	09.01.2026	**Document 1:**<br>('breast reconstruction'/exp OR 'mammaplasty'/exp OR 'free tissue flap'/exp OR 'microsurgery'/exp OR 'reconstructive surgery'/exp OR 'breast reconstruction':ti,ab OR 'autologous breast reconstruction':ti,ab OR 'free flap':ti,ab OR 'free tissue transfer':ti,ab OR 'microvascular reconstruction':ti,ab OR 'microvascular free flap':ti,ab OR 'perforator flap':ti,ab OR diep:ti,ab OR 'deep inferior epigastric perforator':ti,ab OR dieap:ti,ab OR 'deep inferior epigastric artery perforator':ti,ab OR tram:ti,ab OR 'transverse rectus abdominis myocutaneous':ti,ab OR siea:ti,ab OR 'superficial inferior epigastric artery':ti,ab OR sgap:ti,ab OR 'superior gluteal artery perforator':ti,ab OR pap:ti,ab OR 'profunda artery perforator':ti,ab OR tmg:ti,ab OR 'transverse myocutaneous gracilis':ti,ab OR tug:ti,ab OR 'transverse upper gracilis':ti,ab OR 'latissimus dorsi flap':ti,ab OR 'ld flap':ti,ab) AND ('lymphatic surgery'/exp OR 'lymph node transfer'/exp OR 'lymphatic anastomosis' OR 'lymphatic reconstruction'/exp OR 'lymphatic surgery':ti,ab OR 'lymphatic reconstructive surgery':ti,ab OR 'lymphovenous anastomosis':ti,ab OR 'lymphovenous bypass':ti,ab OR lva:ti,ab OR 'vascularized lymph node transfer':ti,ab OR vlnt:ti,ab OR 'lymph node flap':ti,ab OR 'gastroepiploic lymph node transfer':ti,ab OR 'lymph node transplantation':ti,ab) AND ('breast cancer'/exp OR 'breast cancer':ti,ab OR 'breast carcinoma':ti,ab OR mastectomy:ti,ab OR postmastectomy:ti,ab)	Humans, English, 2000–current	n = 157 (Dok1) und 155 (Dok2)

		**Document 2:**<br>('breast reconstruction'/exp OR 'mammaplasty'/exp OR 'free tissue flap'/exp OR 'microsurgery'/exp OR 'reconstructive surgery'/exp OR 'breast reconstruction':ti,ab OR 'autologous breast reconstruction':ti,ab OR 'free flap':ti,ab OR 'free tissue transfer':ti,ab OR 'microvascular reconstruction':ti,ab OR 'microvascular free flap':ti,ab OR 'perforator flap':ti,ab OR diep:ti,ab OR 'deep inferior epigastric perforator':ti,ab OR dieap:ti,ab OR 'deep inferior epigastric artery perforator':ti,ab OR tram:ti,ab OR 'transverse rectus abdominis myocutaneous':ti,ab OR siea:ti,ab OR 'superficial inferior epigastric artery':ti,ab OR sgap:ti,ab OR 'superior gluteal artery perforator':ti,ab OR pap:ti,ab OR 'profunda artery perforator':ti,ab OR tmg:ti,ab OR 'transverse myocutaneous gracilis':ti,ab OR tug:ti,ab OR 'transverse upper gracilis':ti,ab OR 'latissimus dorsi flap':ti,ab OR 'ld flap':ti,ab) AND ('lymphatic surgery'/exp OR 'lymph node transfer'/exp OR 'lymphatic anastomosis' OR 'lymphatic reconstruction'/exp OR 'lymphatic surgery':ti,ab OR 'lymphatic reconstructive surgery':ti,ab OR 'lymphovenous anastomosis':ti,ab OR 'lymphovenous bypass':ti,ab OR lva:ti,ab OR 'vascularized lymph node transfer':ti,ab OR vlnt:ti,ab OR 'lymph node flap':ti,ab OR 'gastroepiploic lymph node transfer':ti,ab OR 'lymph node transplantation':ti,ab) AND ('breast cancer'/exp OR 'breast cancer':ti,ab OR 'breast carcinoma':ti,ab OR mastectomy:ti,ab OR postmastectomy:ti,ab) AND ('lymphedema'/exp OR lymphedema:ti,ab OR lymphoedema:ti,ab OR 'breast cancer related lymphedema':ti,ab OR bcrl:ti,ab)		
**Ovid MEDLINE**	09.01.2026	**Document 1:**<br>(<br>exp Breast Reconstruction/<br>or exp Mammaplasty/<br>or exp Free Tissue Flaps/<br>or exp Microsurgery/<br>or exp Reconstructive Surgical Procedures/<br>or (<br>breast reconstruction<br>or autologous breast reconstruction	Humans, English, 2000–current	n = 133 (Dok1) und 124 (Dok2)

		or free flap<br>or free tissue transfer<br>or microvascular reconstruction<br>or perforator flap<br>or DIEP<br>or "deep inferior epigastric perforator"<br>or DIEAP<br>or "deep inferior epigastric artery perforator"<br>or TRAM<br>or "transverse rectus abdominis myocutaneous"<br>or SIEA<br>or "superficial inferior epigastric artery"<br>or SGAP<br>or "superior gluteal artery perforator"<br>or PAP<br>or "profunda artery perforator"<br>or TMG<br>or "transverse myocutaneous gracilis"<br>or TUG<br>or "transverse upper gracilis"<br>or "latissimus dorsi flap"<br>).ti,ab.<br>)<br>and<br>(<br>exp Lymphatic Vessels/su<br>or exp Lymph Node Transplantation/<br>or (<br>lymphatic surgery<br>or lymphatic reconstructive surgery<br>or lymphovenous anastomosis<br>or lymphovenous bypass<br>or LVA<br>or vascularized lymph node transfer<br>or VLNT<br>or lymph node flap<br>or lymphatic reconstruction<br>or gastroepiploic lymph node transfer<br>or lymph node transplantation		

		).ti,ab.<br>)<br>and<br>(<br>exp Breast Neoplasms/<br>or (<br>breast cancer<br>or breast carcinoma<br>or mastectomy<br>or postmastectomy<br>).ti,ab.<br>)<br><br>**Document 2:**<br>(<br>exp Breast Reconstruction/<br>or exp Mammaplasty/<br>or exp Free Tissue Flaps/<br>or exp Microsurgery/<br>or exp Reconstructive Surgical Procedures/<br>or (<br>breast reconstruction<br>or autologous breast reconstruction<br>or free flap<br>or free tissue transfer<br>or microvascular reconstruction<br>or perforator flap<br>or DIEP<br>or "deep inferior epigastric perforator"<br>or DIEAP<br>or "deep inferior epigastric artery perforator"<br>or TRAM<br>or "transverse rectus abdominis myocutaneous"<br>or SIEA<br>or "superficial inferior epigastric artery"<br>or SGAP<br>or "superior gluteal artery perforator"<br>or PAP<br>or "profunda artery perforator"		

		or TMG<br>or "transverse myocutaneous gracilis"<br>or TUG<br>or "transverse upper gracilis"<br>or "latissimus dorsi flap"<br>).ti,ab.<br>)<br>and<br>(<br>exp Lymphatic Vessels/su<br>or exp Lymph Node Transplantation/<br>or (<br>lymphatic surgery<br>or lymphatic reconstructive surgery<br>or lymphovenous anastomosis<br>or lymphovenous bypass<br>or LVA<br>or vascularized lymph node transfer<br>or VLNT<br>or lymph node flap<br>or lymphatic reconstruction<br>or gastroepiploic lymph node transfer<br>or lymph node transplantation<br>).ti,ab.<br>)<br>and<br>(<br>exp Breast Neoplasms/<br>or (<br>breast cancer<br>or breast carcinoma<br>or mastectomy<br>or postmastectomy<br>).ti,ab.<br>)<br>and<br>(<br>exp Lymphedema/<br>or exp Lymphatic Diseases/su		

		or (<br>lymphedema<br>or lymphoedema<br>or breast cancer related lymphedema<br>or BCRL<br>).ti,ab.<br>)		
**Cochrane Library, CENTRAL**	09.01.2026	**1st row:**<br>breast reconstruction OR autologous breast reconstruction OR free flap OR free tissue transfer<br>OR microvascular reconstruction OR microvascular free flap OR perforator flap<br>OR DIEP OR deep inferior epigastric perforator<br>OR DIEAP OR deep inferior epigastric artery perforator<br>OR TRAM OR transverse rectus abdominis myocutaneous<br>OR SIEA OR superficial inferior epigastric artery<br>OR SGAP OR superior gluteal artery perforator<br>OR PAP OR profunda artery perforator<br>OR TMG OR transverse myocutaneous gracilis<br>OR TUG OR transverse upper gracilis<br>OR latissimus dorsi flap<br>**2nd row**<br>lymphatic surgery OR lymphatic reconstructive surgery<br>OR lymphovenous anastomosis OR lymphovenous bypass<br>OR LVA<br>OR vascularized lymph node transfer OR VLNT<br>OR lymph node flap<br>OR lymphatic reconstruction<br>OR gastroepiploic lymph node transfer<br>OR lymph node transplantation<br>**3rd row:**<br>breast cancer OR breast carcinoma OR mastectomy OR postmastectomy<br>**= Doc 1**<br>**+ 4th row:**<br>lymphedema OR lymphoedema OR breast cancer related lymphedema OR BCRL<br>**= Doc 2**	English, 2000–current	n = 67 (Dok1) und 24 (Dok2)

**Web of Science**	09.01.206	TS=(<br>"breast reconstruction" OR "autologous breast reconstruction" OR mammaplasty<br>OR "free flap" OR "free tissue transfer"<br>OR "microvascular reconstruction" OR "microvascular free flap"<br>OR microsurgery OR microvascular<br>OR "perforator flap"<br>OR DIEP OR "deep inferior epigastric perforator"<br>OR DIEAP OR "deep inferior epigastric artery perforator"<br>OR TRAM OR "transverse rectus abdominis myocutaneous"<br>OR SIEA OR "superficial inferior epigastric artery"<br>OR SGAP OR "superior gluteal artery perforator"<br>OR PAP OR "profunda artery perforator"<br>OR TMG OR "transverse myocutaneous gracilis"<br>OR TUG OR "transverse upper gracilis"<br>OR "latissimus dorsi flap" OR "LD flap"<br>)<br>AND TS=(<br>"lymphatic surgery" OR "lymphatic reconstructive surgery"<br>OR "lymphovenous anastomosis" OR "lymphaticovenous anastomosis"<br>OR "lymphovenous bypass"<br>OR LVA<br>OR "vascularized lymph node transfer" OR VLNT<br>OR "lymph node transfer" OR "lymph node flap"<br>OR "lymphatic reconstruction"<br>OR "gastroepiploic lymph node transfer"<br>OR "lymph node transplantation"<br>OR "immediate lymphatic reconstruction"<br>OR ILR OR LYMPHA<br>OR supermicrosurgery<br>)<br>AND TS=(<br>"breast cancer" OR "breast carcinoma"<br>OR mastectomy OR postmastectomy<br>)	English, 2000–current	n = 223

Table S2. MINORS score.

Study	(1) A clearly stated aim: The question addressed should be precise and relevant in the light of available literature available literature	(2) Inclusion of consecutive patients: All patients potentially fir for inclusion (satisfying the criteria for inclusion) have been included in the study during the study period	(3) Prospective collection of data: Data were collected according to a protocol established before the beginning of the study.	(4) Endpoints appropriate to the aim of the study: Unambiguous explanation of the criteria used to evaluate the main outcome, which should be in accordance with the question addressed by the study. Also, the endpoints should be assessed on an intention-to-treat basis.	(5) Unbiased assessment of the study endpoint: Blind evaluation of objective endpoints and double-blind evaluation of subjective endpoints. Otherwise the reasons for not blinding should b stated.	(6) Follow-up period appropriate to the aim of the study: The follow-up should be sufficiently long to allow the assessment of the main endpoint and possible adverse events.	(7) Loss to follow up less than 5%: All patient should be included in the follow up. Otherwise, the proportion lost to follow up should not exceed the proportion experiencing the major endpoint.	(8) Prospective calculation of study size: Information of the size of detectable difference of interest with a calculation of 95% confidence interval, according to the expected incidence of the outcome event, and information about the level for statistical significance and estimates of power when comparing outcomes.	**MINORS Score**
**Szymanski (2024)**	NA	NA	NA	NA	NA	NA	NA	NA	NA
**Aktia (2016)**	NA	NA	NA	NA	NA	NA	NA	NA	NA
**Akita (2017)**	2	0	0	1	0	2	0	0	5
**Parikh (2021)**	NA	NA	NA	NA	NA	NA	NA	NA	NA

**Demiri (2024)**	0	2	2	2	1	2	0	0	9
**Myung (2023)**	2	2	0	2	0	2	0	0	8
**Dionyssiou (2021)**	2	0	0	1	1	2	1	0	7
**Dionyssiou (2022)**	2	0	0	1	1	2	1	0	7
**Chang (2020)**	2	0	0	1	1	1	1	0	6
**Di Taranto (2023)**	2	0	0	1	0	2	0	0	5
**Winters (2022)**	2	0	0	2	0	2	2	0	8
**De Brucker (2016)**	2	0	0	2	0	2	2	0	8
**Nguyen (2015)**	2	1	0	1	1	1	1	0	7
**Dancey (2013)**	1	1	0	1	1	1	1	0	6
**Saaristo (2012)**	1	1	0	1	0	0	0	0	3
**Wallis (2019)**	1	0	0	0	0	0	0	0	1
**Chen (2014)**	1	0	0	1	0	0	0	0	2
**Chu (2023)**	1	0	0	0	0	0	0	0	1
**Chang (2018)**	2	1	2	1	1	2	0	0	9
**Chang (2020)**	2	2	2	2	1	1	0	0	10
**Montag (2019)**	1	1	2	2	1	1	2	0	10
**Ciudad (2022)**	NA	NA	NA	NA	NA	NA	NA	NA	NA
**Crowley (2024)**	1	1	0	0	0	1	0	0	3
**Ciudad (2020)**	1	1	0	0	0	0	0	0	2
**Ciudad (2023)**	2	1	0	2	0	2	0	0	7

**Engel (2018)**	2	1	2	1	1	2	2	0	11
**Deldar (2017)**	1	1	0	0	0	1	0	0	3
